# A Novel Method of Aircraft Detection Based on High-Resolution Panchromatic Optical Remote Sensing Images

**DOI:** 10.3390/s17051047

**Published:** 2017-05-06

**Authors:** Wensheng Wang, Ting Nie, Tianjiao Fu, Jianyue Ren, Longxu Jin

**Affiliations:** 1 Changchun Institute of Optics, Fine Mechanics and Physics, Chinese Academy of Sciences, Changchun 130033, China; nieting89@163.com (T.N.); futj86@yeah.net (T.F.); renjyue@163.com (J.R.); jinlongx@yeah.net (L.J.); 2 University of Chinese Academy of Sciences, Beijing 100049, China

**Keywords:** pattern recognition, active contours, convex hull detection, target detection

## Abstract

In target detection of optical remote sensing images, two main obstacles for aircraft target detection are how to extract the candidates in complex gray-scale-multi background and how to confirm the targets in case the target shapes are deformed, irregular or asymmetric, such as that caused by natural conditions (low signal-to-noise ratio, illumination condition or swaying photographing) and occlusion by surrounding objects (boarding bridge, equipment). To solve these issues, an improved active contours algorithm, namely region-scalable fitting energy based threshold (TRSF), and a corner-convex hull based segmentation algorithm (CCHS) are proposed in this paper. Firstly, the maximal variance between-cluster algorithm (Otsu’s algorithm) and region-scalable fitting energy (RSF) algorithm are combined to solve the difficulty of targets extraction in complex and gray-scale-multi backgrounds. Secondly, based on inherent shapes and prominent corners, aircrafts are divided into five fragments by utilizing convex hulls and Harris corner points. Furthermore, a series of new structure features, which describe the proportion of targets part in the fragment to the whole fragment and the proportion of fragment to the whole hull, are identified to judge whether the targets are true or not. Experimental results show that TRSF algorithm could improve extraction accuracy in complex background, and that it is faster than some traditional active contours algorithms. The CCHS is effective to suppress the detection difficulties caused by the irregular shape.

## 1. Introduction

Remote sensing images contain large amount of geo-graphical environmental information and have widely used in different scientific fields. Target detection (e.g., bridges, ships and aircrafts) is one of the fundamental applications in remote sensing images. It is an important basis to conduct military attack and intelligent transportation, rapidly and correctly [1,2]. Among these targets, aircraft detection becomes a research hotspot due to its important military and traffic status [3,4,5,6,7,8,9,10,11,12,13,14]. With the resolution improvement of optical remote sensing image, the abundant detail information of targets makes target detection more easily. However, some intractable obstacles, including the hugeness and complication of spatial data, changeable environment and relatively low signal-to-noise ratio (SNR), cause the low detection efficiency and accuracy.

Specifically, there are two major challenges that hinder the improvement of the detection effect:

One is that how to extract targets exactly from the complex background. For this challenge, there are few systematic methods to cope with it. A suspicious target is usually extracted from the thumbnail slice containing the well-marked target. Arandjelović [3] proposed an efficient and accurate set-based registration algorithm of aerial images through constraint graphs optimization of pairwise image registration. They formulate the joint registration problem as an optimization scheme. Although this idea is very novel, it needs to be further studied before the application to the target detection area.

The other one is identifying the targets in case of irregular target shapes target due to natural conditions (low signal-to-noise ratio, illumination condition or swaying photographing) and occlusion by surrounding objects (boarding bridge, equipment). This part could be classified into two major strands. One strand is to take advantage of the shape of an aircraft. With the aid of the obvious structural features, including principal axis, contour and rigidity, the scholars extracted and detected the aircrafts by symmetry and various rotation-invariant moments of aircrafts. Some classical algorithms are combined to be used, such as principle component analysis, Zernike invariant moments, contour tracking and so on [4,5,6,7,8]. These methods always extract rotation-invariant or symmetrical features from an overall contour obtained by gray value threshold segmentation, which means the extracted contour should be perfect and regular. However, the idealized objects make these methods fail when the target is deformed, irregular or asymmetric due to low SNR, optical aberrance or swaying photographing. Arandjelović [9] proposed a gradient edge mapping algorithm for face recognition. The vertical symmetry was used in the edge extraction process, which may be applied to symmetry target detection in remote sensing image. The other strand focuses on building a target sample library to match the targets. Because of the uniqueness of aircraft structure, few false-alarm targets can be matched the aircraft structure. For example, Cheng et al. [11] extract the gradient direction of targets through the HOG operator and obtain the aircraft targets by matching the trained samples to the region of interest (ROI). The aircrafts gradient direction feature is not unique, leading to the large number of false targets in the preliminary processing, and increasing the burden of subsequent processing. Moreover, it is hard to establish the sample library that contains all the types of aircrafts. Facing the difficulty of establishment of the sample library, Liu et al. [12] match the targets by calculating an average template based on a large number of samples and identify the targets in accordance with their similarity to the template. But such method also needs a lot of samples for training, and the average template could not match all the targets. In summary, the above-mentioned methods all focus on the whole targets in the small and clean image slice. Arandjelović [13] proposed a method of object matching for smooth and untextured images using boundary descriptors, which comprises a profile of sampled boundary normals’ directions. The equidistant key points are obtained by sampling the image boundary curves. Then, the boundary normals of a local profile around one key point were utilized to describe this point. The proposed method was shown to be robust in many classical object image sets. But the local descriptor is more sensitive to local noise and incorrect shape components, which commonly exist in remote sensing images.

In this paper, we solve the above-mentioned problems in two steps. Unlike the ocean target detection, the remote sensing image of land consists of many gray levels, which causes relative difficulty to extract the candidates from the image utilizing pure threshold segmentation, e.g., the popular Otsu’s algorithm [15]. Therefore, an improved active contour method is provided for ROI extraction. To ensure fast operation and accurate segmentation, the proposed approach combines Otsu’s algorithm with active contour to segment the images. Meanwhile, the aircraft is a rigid target with fixed structure, a special shape and salient corners. According to these characteristics, we propose an algorithm named corner-convex hull based segmentation algorithm (CCHS), and it can effectively overcome the problem of irregular aircraft confirmation. The algorithm analyzes contour features of an aircraft, extracts the aircraft corners, draws the minimum convex hull via extracted corners, and divides the convex hull into several fragments. A series of new features named target-to-fragment radio (TFR) and fragment-to-hull radio (FHR) obtained from the segmented targets can help the targets identification by support vector machine (SVM) [16]. The advantages of the present approach include (1) improving the precision of the aircraft ROI extraction in the complex background; (2) improving the detection precision of the irregular shape aircraft after ROI extraction. Experiments on Google Earth images show that the proposed method is effective and robust in complex and large sense.

## 2. Detection Algorithm

### 2.1. A Whole Process

**Figure 1:**
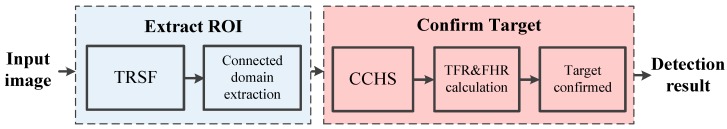
Proposed aircraft detection algorithm framework.

**Figure 2:**
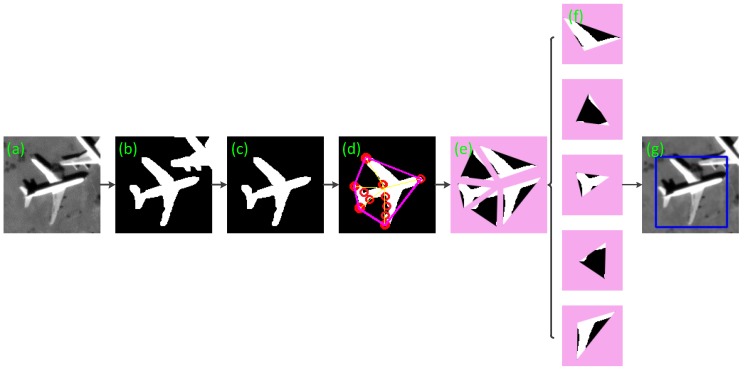
Diagrammatic sketch of target detection process for an aircraft example. In the figure, (**a**) is about the input, (**b**) is about the binarization, (**c**) is regarding the extracted connected regions, (**d**) is for determination of corner convex hull, (**e**) is the segmentation effect, (**f**) shows each fragment, and (**g**) gives the final results.

The proposed detection algorithm is divided into two steps, as shown in the Figure 1. In the stage of extracting candidate area, the input image is binarized through the TRSF algorithm, and the binary image is extracted the connected region after morphological treatment. In the stage of target identification, the CCHS algorithm checks the convex hull via the corners of connected regions, and divides the targets into fragments. At last, the fragments are analyzed to confirm the targets and output the results. Figure 2 gives an intuitive illustration by an example.

### 2.2. ROI Extraction

The binarization of the input image is a necessary operation before extracting the connected regions. In the Otsu’s algorithm, the segmentation threshold is obtained through the gray histogram calculation based on statistical information. Such algorithm is characterized by simple, stable and effective calculations and ensures the image segmentation quality in the case of simple background. It is usually regarded as one of the most fashionable threshold segmentation algorithms. However, considering that a remote sensing image consists of numerous grey levels, it seems a difficult task to extract the ROI from such a complex image directly through Otsu’s algorithm. It is found out that the aircraft luminance is usually very high through observation. So the suspected aircraft area can be obtained through further gray histogram segmentation based on Otsu’s algorithm pre-segmentation. The active contour algorithm, such as C-V model [17], can effectively segment a target with a weak boundary. But the conventional C-V model has low computational efficiency with many iterations, and it is unable to process the case of many gray levels. By approaching its energy function curve to the target contour, the Region-Scalable Fitting (RSF) model [18] can segment the image with uneven intensity distribution. But such model is sensitive to the contour line. Simultaneously, the initial contour has certain effects on both the number of iterations and convergence. By combining the merits of Otsu’s algorithm and RSF, this paper proposes the improved segmentation algorithm TRSF for target segmentation and extraction.

The Otsu’s algorithm characterizes the separating properties of both the targets and the background through the Interclass Variance, which is defined as:

(1) $$\sigma^{2}\left(T\right)=W_{a}{\left(\mu_{a}-\mu \right)}^{2}-W_{b}{\left(\mu_{b}-\mu \right)}^{2},$$

where $\sigma^{2}$ is the variance between two classes, $W_{a}$ is the proportion of Class A in the image, $\mu_{a}$ is the gray average of Class A, $W_{b}$ is the proportion of Class B in the image, $\mu_{b}$ is the gray average of Class B, and μ is the overall gray average of the image. The T, which maximizes the total interclass variance $\sigma^{2}(T)$, is just the segmentation threshold between the minimum and maximum gray values. The segmented result is defined as level set function ϕ as follows.

(2) $$\phi (x)=\left\{ \begin{array}{l} 1\;，x\in \Omega_{1}\\ -1，x\in \Omega_{2} \end{array}\right.,$$

where $\Omega_{1}$ is the segmented quasi-target region, and $\Omega_{2}$ is the quasi-background region. The energy function of RSF model in the level set is:

(3) $$\{\begin{array}{l} E=\sum_{i=1}^{2}\lambda_{i}\int_{\Omega }\int_{\Omega }K_{\sigma }\left(x-y\right){\left|I\left(y\right)-f_{i}\left(x\right)\right|}^{2}M_{i}\left(\phi \left(y\right)\right)dydx\\ L\left(\phi \right)=\int_{\Omega }\left|\nabla H\left(\phi \left(x\right)\right)\right|dx\\ P\left(\phi \right)=\int_{\Omega }\frac{1}{2}{\left(\left|\nabla \phi \left(x\right)\right|-1\right)}^{2}dx \end{array},$$

Equation (3) is composed of three terms, where L(ϕ) is the length of closed contour line, P(ϕ) is the regular term of level set, and $\lambda_{i}$ is a non-negative constant representing the weighting coefficient of every energy term. $M_{1}(\phi )=H(\phi )$ and $M_{2}(\phi )=1-H(\phi )$. The H(ϕ) is approximated to Heaviside function:

(4) $$H_{\varepsilon }(x)=\frac{1}{2}\left[1+\frac{2}{\pi }\arctan\left(\frac{x}{\varepsilon }\right)\right],$$

The introduced non-negative kernel function $K_{\sigma }$ is defined as:

(5) $$K_{\sigma }(u)=\frac{1}{2\pi \sigma^{2}}\exp(\frac{-{\left|u\right|}^{2}}{2\sigma^{2}}),$$

where σ is positive scale parameter.

When the closed boundary ϕ is not located in the two homogeneous regions, the minimum of E cannot be achieved. The variable E gets its minimal value if the contour line is on the boundary between two homogeneous regions. Therefore, the two matching functions $f_{1}(x)$ and $f_{2}(x)$ are adopted to fit the image intensity of the areas on both sides of the boundary ***C***, which is defined as:

(6) $$f_{i}(x)=\frac{K_{\sigma }(x)\times \left[M_{i}(\phi (x))I(x)\right]}{K_{\sigma }(x)\times M_{i}(\phi (x))},$$

The introduced kernel function $K_{\sigma }$ can control the boundary scalability, which makes the most of local intensity information of an image. This model segments an image with intensity inhomogeneity and could achieve relatively good segmentation results for some objects with weak boundaries.

**Figure 3:**
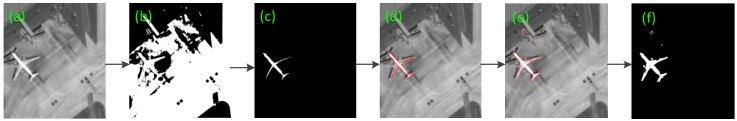
Extraction of image target region: (**a**) Original image; (**b**) the first Otsu’s algorithm segmentation; (**c**) the second segmentation; (**d**) Initial RSF boundary; (**e**) RSF result; (**f**) Binary segment result.

This paper employs in coarse-to-fine fashion to extract the ROIs. Figure 3 shows this process in detail as follows:

- Downsample the original image by a factor of 4, and then implement initial Otsu’s algorithm segmentation for the low-resolution image. In this way, the complexity of computation is reduced.
- After the completion of Otsu’s algorithm, weed out small areas at first, and then process the segmented region through secondary segmentation and morphological processing at a high gray level (10%). At this point, the aircraft area can be located basically.
- Take the segmented binary image as the local region and initial condition of active contour, define the initial level set function ϕ as the piecewise function containing only two function values, namely 1 and −1, and use this function to guide the curve evolution in the image.
- Evolve the level set function through energy functionality, and search for the level set K={x|ϕ(x)≤0}. When the symbol of ϕ(x) changes, the corresponding energy functionality will decrease so that ϕ(x)=−ϕ(x). Otherwise, keep ϕ(x) unchanged.
- Repeat the steps c and d, and stop the repetition when the energy functionality doesn’t change any more. Finally, the segmentation result can be obtained.
- Process the segmented binary image morphologically, and weed out small areas.

For subsequent processing, we employ the searching algorithm for 8-neighborhood connected regions to extract the connected regions from a binary image [19]. Suppose the image function is f(x,y), the (p+q)-order moment is given as:

(7) $$m_{pq}=\overset{M}{\underset{x=1}{\sum }}\overset{N}{\underset{y=1}{\sum }}x^{p}y^{q}f(x,y),\;p,q=0,1,2\ldots$$

The area is the zero-order moment, $\left(\bar{x},\bar{y}\right)$ is the centroid of a connected region, and $\bar{x}=m_{10}/m_{00}$, $\bar{y}=m_{01}/m_{00}$.

### 2.3. ROI Analysis to Confirm Target

In this section, a corner-convex-hull based segmentation (CCHS) algorithm is given. Furthermore, the new features—namely TFR and FHR—are introduced into the judgment of true targets particularly.

#### 2.3.1. Corner-Convex-Hull Based Segmentation Algorithm

**Figure 4:**
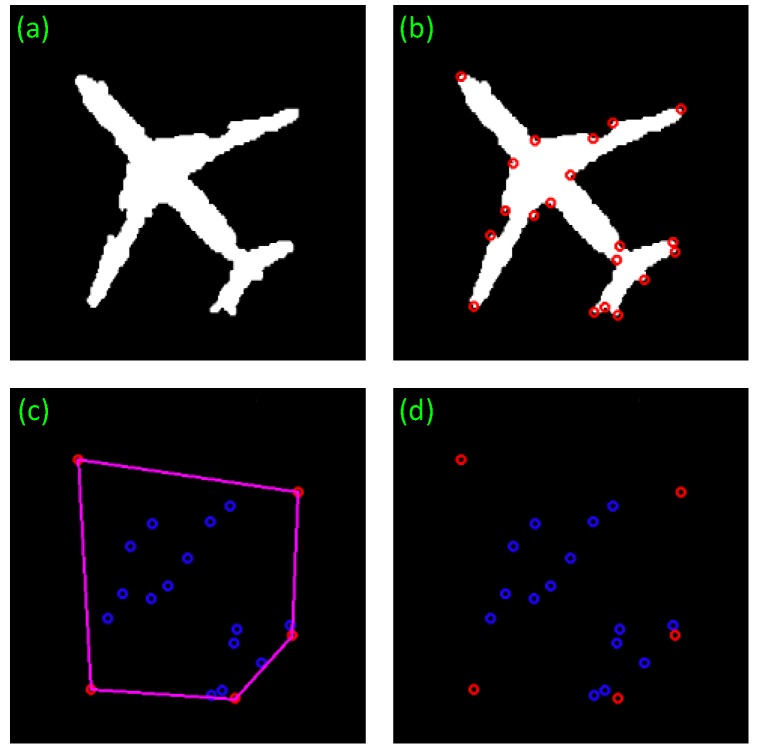
Corner-convex hull detection result. (**a**) The connected region of aircraft in the Figure 3f. (**b**) The Harris points of the connected region. (**c**) The blue points are the interior points. (**d**) The result of convex hull detection of these points.

After the last step, many of the connected regions, which include the connected regions of the aircraft target, have been extracted by TRSF algorithm. Then we would exclude the false targets in these connected regions. The aircraft targets should be filtered in accordance with the aircraft features that we presented below. The most fundamental features, such as area and aspect ratio of the minimum external moment, are used to eliminate a large number of regions. Since the aircraft contour takes on typical geometric features, its feature points are available for target screening. With a small calculation burden and steady results, the Harris corner operator [20] is adopted. Corners are important local features of a target because they cannot only keep important shape information of a target, but also reduce redundant data of the target effectively to relieve the computation burden. Besides, corners remain invariant during transition, zooming and rotation. Thus, corner feature is the crucial basis for aircraft detection method proposed by this paper. The response value *R* of Harris corner points is:

(8) $$R=\det(C)-k\times {\mathrm{trace}}^{2}(C),$$

where $C\left(x\right)=\left[\begin{array}{cc} I_{u}^{2}\left(x\right) & I_{uv}\left(x\right)\\ I_{uv}\left(x\right) & I_{u}^{2}\left(x\right) \end{array}\right]$, in which $I_{u}\left(x\right)$, $I_{v}\left(x\right)$, and $I_{uv}\left(x\right)$ are the partial derivatives and second-order mixed partial derivative of the gray value of image point x in the R and v directions; k is an empirical value, usually between 0.04~0.06 and we set 0.04. By setting the threshold T, a point whose response value R is bigger than T is considered as Harris corner. The detection result of target corners is shown in the Figure 4b.

After the extraction of corners from a segmented binary image, it is found that an aircraft has five salient corners at the aircraft nose, wings and tail respectively. Convex hull [21] of these corners reveals that the convex hull of aircraft corners is a pentagon constituting only five vertexes or corners at the aircraft nose, wings and tail respectively, while other corners are located inside the convex hull, as shown in Figure 4c. Some defective corners caused by segmentation are also found inside the convex hull. Hence, some defects arising from threshold segmentation have no effects on the extraction of convex hull. The corner extraction from a binary image can also shorten the operation time.

When multiple adjacent corners overlap, the similar corners can be merged in accordance by applying the Euclidean distance to prevent one feature point from being detected as several corners. In this way, the pentagon corners for an aircraft become extremely clear. The usage of extracted corner set can greatly reduce the calculation burden in convex closure detection in a whole connected region.

The process of convex hull detection in this section is given as follows (Algorithm 1):
**Algorithm 1**
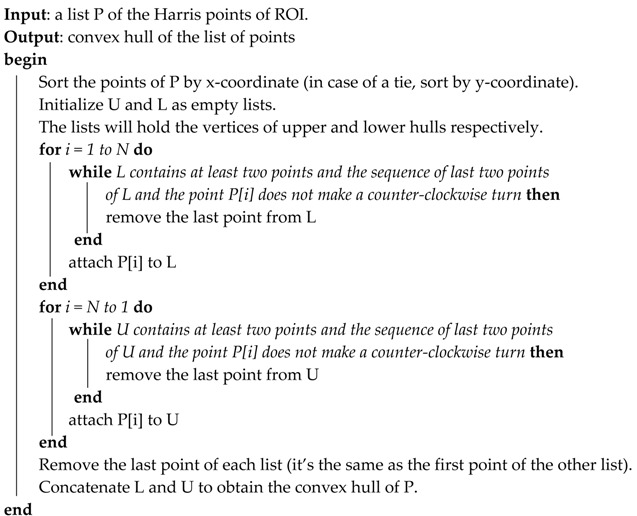



Points in the result will be listed in counter-clockwise order.

#### 2.3.2. New Features for Target Confirmation

After drawing the convex hull of ROIs, there are still some false candidates in a pentagon shape which needs to be further wed out. Here, we propose a segmentation approach that divides a connected region into several parts so that every slice can be independently analyzed to remove the false targets.

In this section, the centroid of a connected region is connected to the vertexes of convex hull to segment the region into five fragments. The shape of each fragment is a triangle which includes a part of connected region and a part of convex hull background. The new feature TFR describes the radio of the area of each target part to each triangle and the other feature FHR describes the radio of area of each triangle to the whole hull. The definitions are as follows.

(9) $$TFR_{i}=\frac{S_{target}}{S_{fragment}},$$

(10) $$FHR_{i}=\frac{S_{fragment}}{S_{convex}},$$

where $S_{target}$ is the number of the pixels in a fragment whose value is 1, $S_{fragment}$ is the number of pixels in every whole fragment, and $S_{convex}$ is the number of pixels in the whole fragment.

**Figure 5:**
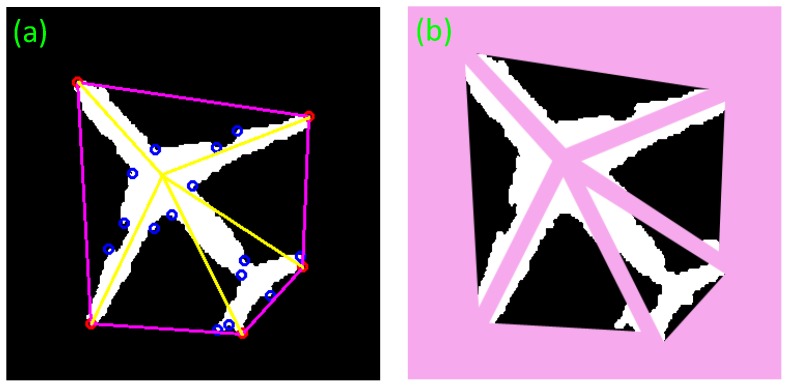
Aircraft fragment segmentation. (**a**) The target is marked with corners and convex hull. The pink pentagon hull is the convex hull of these corners. The blue corners are the internal corners and the red corners are the external corners. The yellow lines are the dividing lines. (**b**) The target is divided into five fragments by the dividing lines.

The result of fragment segmentation is shown in Figure 5. Moreover, Table 1 gives the features of aircraft fragments. Through the five features, an aircraft target can be identified.

According to Table 1, fragments 2 and 4 have a very small TFR of around 10%, followed by the TFR of fragments 1 and 5, which is about 70%. The TFR of fragment 3 is more than 80%, the highest among them. Some regularities can also be found in the distribution of FHR. By adopting these features, false alarms can be rejected.

**Figure 6:**
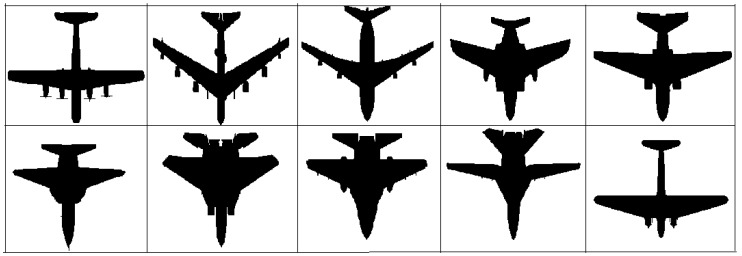
Different aircraft models.

**Figure 7:**
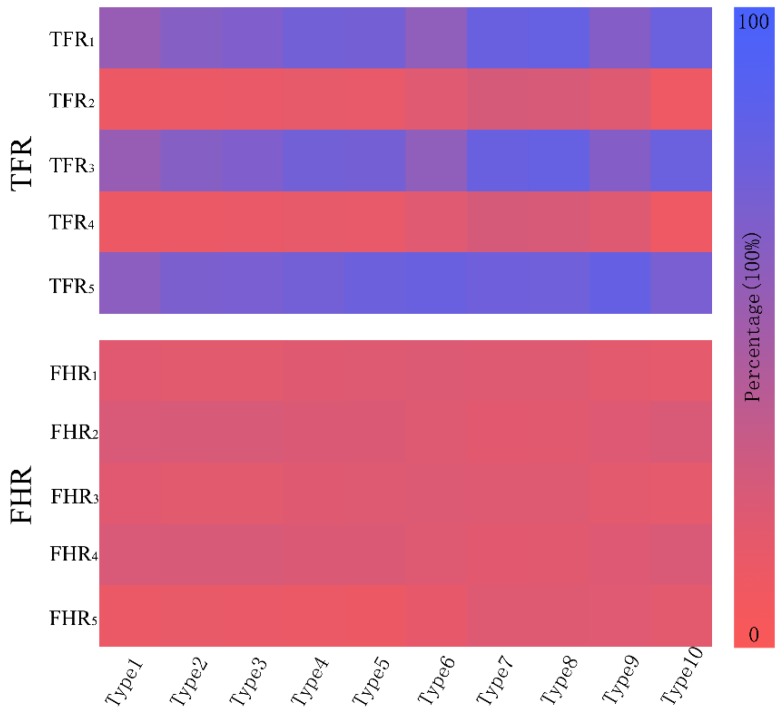
The pseudo color map of TFR and FHR. For different types of aircraft, each feature has few changes. For TFR_i_, the value of TFR_1_, TFR_3_, and TFR_5_ are relatively large; contrarily, the value of TFR_2_ and TFR_4_ are relatively small. For FHR_i_, they are relatively average with a small variance.

The fragment features are applied to different aircraft models [22] shown in the Figure 6, and the corresponding data are shown in the Table 2. It is found that the two features of different aircraft models show some regularity. The pseudo color map shows the regularity more obviously in Figure 7.

**Figure 8:**
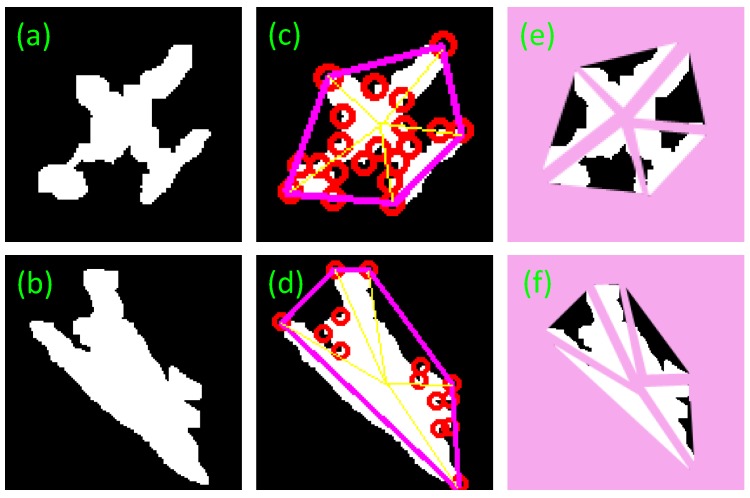
The example of irregular shape aircraft and false alarm from the connected region of Google Earth (**a**) Aircraft defect; (**b**) False alarm; (**c**) Detection results of aircraft defect; (**d**) Detection results of false alarm; (**e**) Segmentation result of aircraft defect; (**f**) Segmentation results of false alarm.

If the extracted aircraft is irregular, as shown in Figure 8a, the invariant moment based on symmetry will be no longer valid. The features proposed in this paper could cope with this problem. Figure 8b illustrates that the false alarm can be rejected though it also has five vertexes.

Through the SVM learning and training for a large number of remote sensing aircraft image slices, the targets can be judged accurately [23].

## 3. Test Process and Results

Due to the lack of standard data sets of high resolution remote sensing images for object detection, we evaluate the proposed method on 52 images from Google Earth for aircraft detection with the resolution of 60 cm/pixel. (Since we concentrates on the aircraft detection in panchromatic image, we convert the RGB Google Earth images into grayscale images for the experiments). We collect 500 positive patches from the Google Earth to train the TFR and FHR features. Simulation is done with Visual Studio 2012 in a computer with a CPU of Intel 4 GHz and 32 G memory to evaluate the calculation time.

### 3.1. Datasets and Experimental Settings

**Figure 9:**
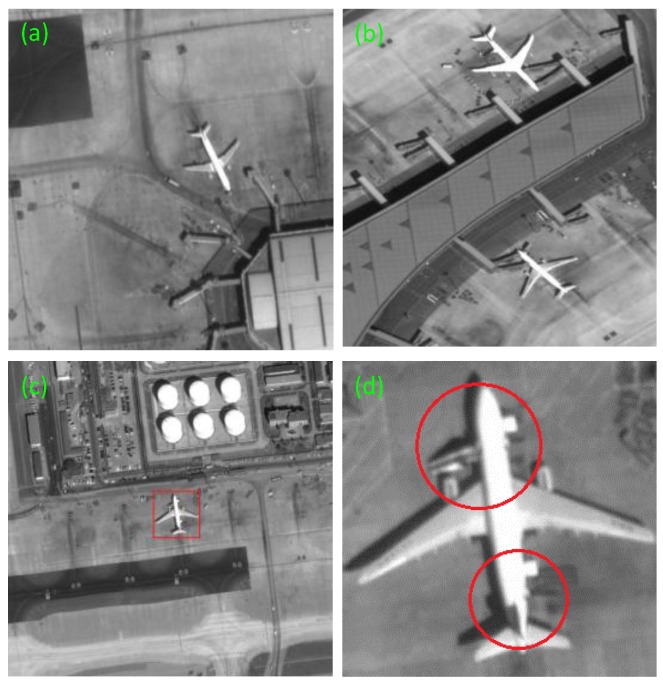
The original images. (**a**) A normal image. (**b**) Two aircrafts with swaying photographing. (**c**) An aircraft in red rectangle is connected together with something others, which can be seen in the enlarged drawing (**d**). (**d**) The superfluous parts are shown in red circles of (**c**). Their sizes are respective 830 × 868, 1183 × 1631, and 2060 × 1992.

As shown in Figure 9, we choose three representative images among the numerous images to show the application of our method.

### 3.2. ROI Extraction

**Figure 10:**
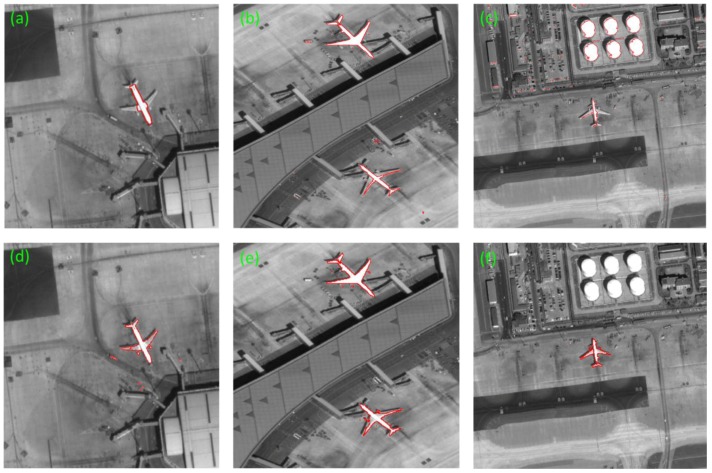
ROI extraction results: (**a**–**c**) the initial RSF contours obtained by threshold segmentation; (**d**–**f**) the final RSF contours after energy function stability.

In this part, we show the result of the ROI extraction using our proposed method. According to [17], we use the following parameters in this paper: σ=3.0, time step Δt=0.1, μ=1, and ν=0.001×255×255. The results of the three images shown in the Section 3.1 are given in the Figure 10.

**Figure 11:**
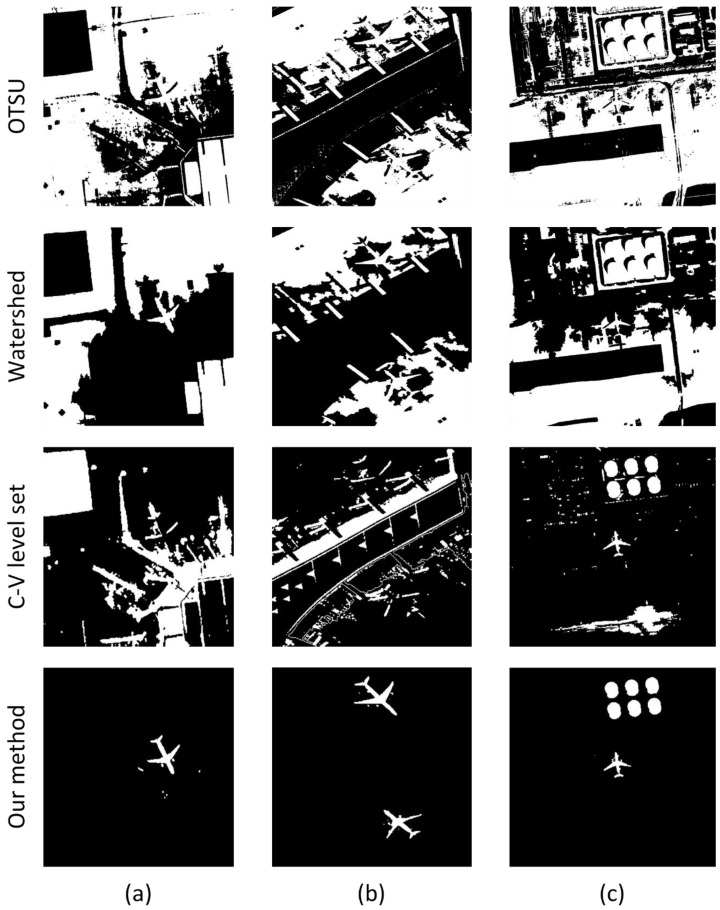
Comparison of segmentation results of different methods. The column (**a**–**c**) results correspond to the original images of Figure 9a–c.

Then, the segmentation algorithms of Otsu’s algorithm, watershed, and C-V level set are compared with our proposed method. Figure 11 shows that the results of our method are significantly superior to some other algorithms because of its high accuracy. Table 3 shows the TRSF’s operation speed is faster than conventional C-V model algorithm. Although it runs slower than the gray value threshold algorithm and watershed algorithm, the segmentation effect is obviously better than the two previous algorithms.

### 3.3. Target Identification

After the ROI segmentation, the corners are detected and their convex hulls are drawn. Then the ROI is segmented into several fragments and the features are extracted. That we adopt the Harris points to calculate the convex hull can greatly improve the running speed compared with the method based on the whole contour. Table 4 shows the time and efficiency of convex closure detection based on the corner points and a whole contour respectively. It is evident that the detection efficiency based on the corners is higher.

**Figure 12:**
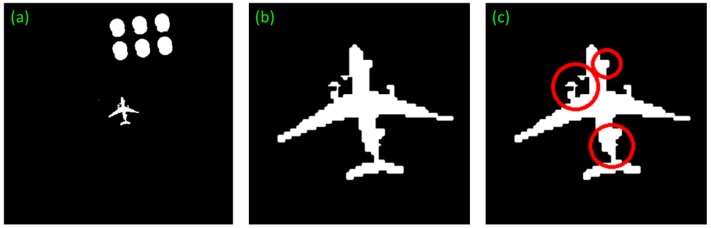
The local enlarged drawing of the Figure 11c. (**a**) is the original images. (**b**) is the local enlarged drawing. (**c**) marks the irregular parts by red circles.

**Figure 13:**
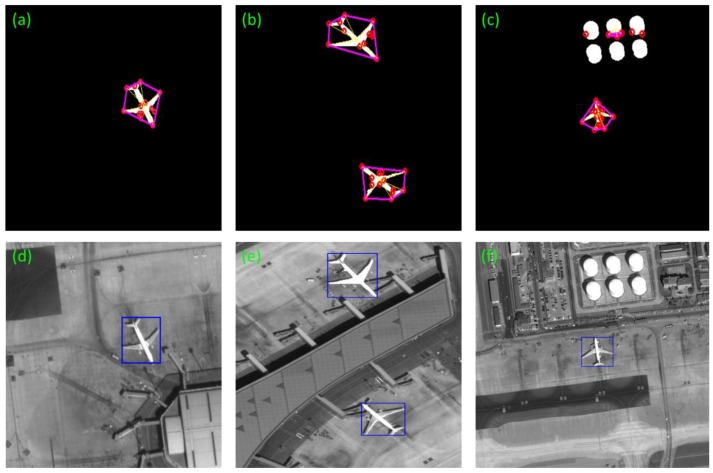
Detection results (continued from Figure 9 of the Section 3.1): (**a**–**c**) are convex hull results of ROIs. (**d**–**f**) are the detection results.

In details, the local enlarged drawing of the Figure 11c of our method in segmentation stage is shown in the Figure 12. The irregular parts are signed by red circles. Our method can correct this defect and detect the targets successfully. Moreover, the swaying photographing image in Figure 8b can also detect accurately. The detection effect is shown in Figure 13.

### 3.4. Conclusion Remarks of Simulations

After the results of target detection output, we evaluate the performance metrics in terms of precision and recall, denoted as *P* and *R* [24]. The precision is aimed to maximize the target detection without missing any target, so the detection rate is the most important criterion in the detection result assessment. The recall is the percentage of false targets in the detection results. The lower the false alarm rate is, the better the algorithm is. As we can see in Table 5, A is the number of images correctly classified as having airports, B denotes the number of images wrongly classified as having airports, and C represents the number of images wrongly classified as having other objects.

*P* and *R* are respectively defined as:

(11) $$P=\frac{A}{A+B},$$

(12) $$R=\frac{A}{A+C},$$

**Figure 14:**
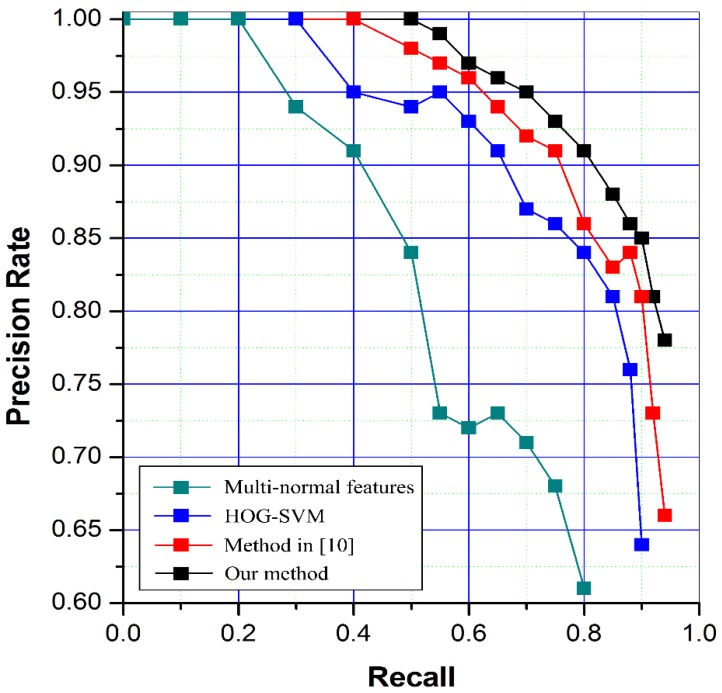
Precision–recall curves of different methods.

**Figure 15:**
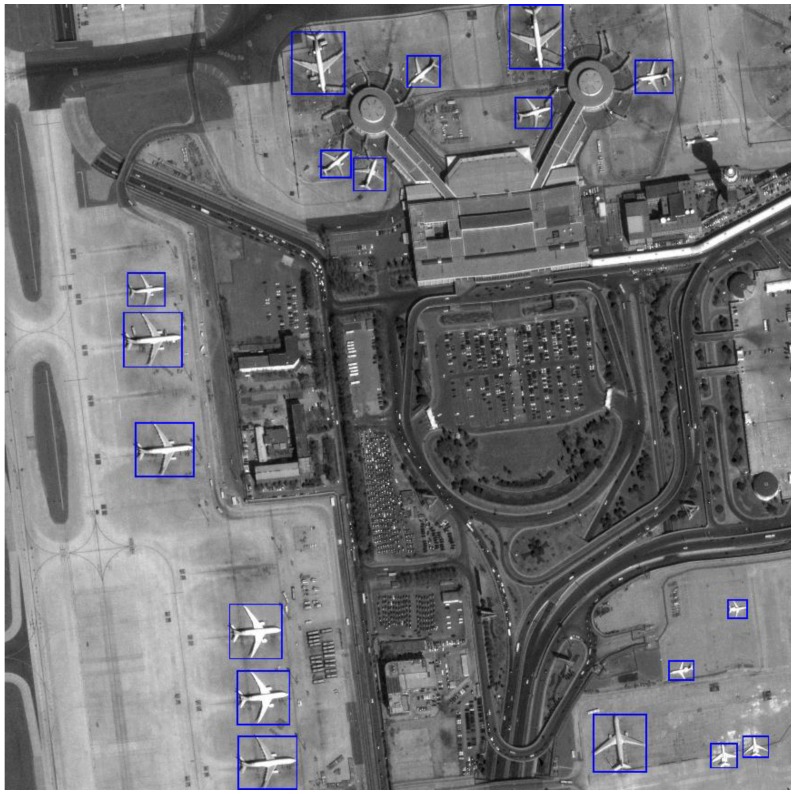
The detection result of an image form Google Earth. The size of the image is 4096 × 4096.

In order to verify the proposed method has a positive impact on aircraft detection, a wide range of tests are conducted on a large number of images involving 312 aircrafts in total. In comparison, a method based on size and Hu moments multi-normal features and a method based on HOG-SVM are used as comparison. All of the methods extract the ROIs using our first stage method and then validate the results in different features. In addition, the method in [10] is also used as comparison to evaluate the proposed method. The recall and precision of the four different methods in total are listed in Table 6. According to the table, it is obvious that the algorithm proposed by this paper has a better detection effect with higher recall and precision. However, the average processing time is not small, mainly because of the iteration of energy function method used for ROI extraction. Overall, the proposed method is effective and robust. And there is room to improve the processing speed, although it is much better than traditional method. The compare result is illustrated in Figure 14 by using precision–recall curves. At last, a result image is given in Figure 15 for exhibition.

## 4. Conclusions

In summary, a novel method has been proposed in two steps to settle the problem of low aircraft detection precision in high-resolution panchromatic optical remote sensing images. First of the two steps, an improved RSF algorithm has been suggested to solve ROI extraction difficulty in complex background. Then, a corner-convex-hull based segmentation algorithm has been introduced to solve the aircraft shape irregularity problems. The new features, target-to-fragment radio (TFR) and fragment-to-hull radio (FHR), have been constructed to take into the false targets elimination. The performance has been evaluated and validated in terms of precision and recall using the images from Google Earth. As a conclusion, it can be observed that the proposed method is tractable to be built and implemented to improve the accuracy.

The further research is underway in the following two directions.

- It is of interest to extend the complex remote sensing background of the target extraction from the aircraft mutatis mutandis to other targets by introducing new feature descriptors, for instance vehicles or marine vessels.
- We are interested in how to combine the panchromatic with multi-spectral information to detect the aircraft target. It is promising to use the high resolution of panchromatic images and the different reflectivity of different spectral bands for different objects to accurate detect targets.

## Figures and Tables

**Table 1:** Fragment features.

Features	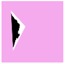	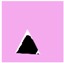	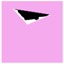	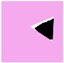	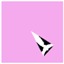
TFR	73.6%	10.3%	73.8%	9.8%	80.5%
FHR	23.4%	19.8%	23.3%	19.6%	13.9%

**Table 2:** Fragment feature data of different aircraft models shown in Figure 6. For the two series of features of each type aircraft in longitudinal data, each of these features is stable, and there is little change among the different types of models. TFR1, TFR3 and TFR5 fluctuate about from 50% to 80%. TFR2 and TFR4 fluctuate about from 10% to 30%. Each of FHRs are also stable, about from 10% to 25%.

Feature	Target-to-Fragment Radio					Fragment-to-Hull Radio				
Number	TFR_1_	TFR_2_	TFR_3_	TFR_4_	TFR_5_	FHR_1_	FHR_2_	FHR_3_	FHR_4_	FHR_5_
**Type 1**	55.3%	10.8%	55.3%	10.8%	60.3%	19.8%	23.9%	19.8%	23.9%	12.6%
**Type 2**	61.5%	12.4%	61.5%	12.4%	68.5%	18.8%	24.0%	18.8%	24.0%	14.4%
**Type 3**	65.4%	12.5%	65.4%	12.5%	69.4%	18.8%	24.4%	18.8%	24.4%	13.6%
**Type 4**	72.1%	14.9%	72.1%	14.9%	72.1%	20.1%	23.2%	20.1%	23.2%	13.4%
**Type 5**	71.5%	15.6%	71.5%	15.6%	75.5%	20.4%	23.2%	20.4%	23.2%	12.8%
**Type 6**	58.6%	19.8%	58.6%	19.8%	77.2%	21.4%	20.8%	21.4%	20.8%	15.6%
**Type 7**	78.6%	25.4%	78.6%	25.4%	74.6%	20.5%	19.1%	20.5%	19.1%	20.8%
**Type 8**	79.2%	24.3%	79.2%	24.3%	74.5%	20.4%	19.3%	20.4%	19.3%	20.6%
**Type 9**	63.2%	20.1%	63.2%	20.1%	79.7%	18.3%	21.5%	18.3%	21.5%	20.4%
**Type 10**	77.8%	10.2%	77.8%	10.2%	69.3%	17.2%	23.5%	17.2%	23.5%	18.6%

**Table 3:** Comparison of segmentation speeds (unit: s).

Number	Size	Otsu’s Algorithm	Watershed	C-V Level Set	Proposed Algorithm
**a**	830 × 868	0.013	0.892	20.126	1.134
**b**	1183 × 1631	0.028	1.548	60.574	2.143
**c**	2060 × 1992	0.052	2.056	100.562	3.289

**Table 4:** Comparison of convex hull detection speeds (unit: ms).

Number	Traditional Convex Hull	Proposed Algorithm
**1**	53	0.6
**2**	119	3.5
**3**	238	1.2

**Table 5:** Relationships among the detection results.

	Related	Unrelated
**Detected**	A (true positives)	B (false positive)
**Undetected**	C (false negative)	–

**Table 6:** Relationships among the detection results.

Method	Precision/%	Recall/%	Average Processing Time/s
**Multi-normal features**	81.3	72.1	2.89
**HOG-SVM**	86.7	83.3	3.58
**Method in [10]**	87.9	85.6	1.94
**Our method**	90.9	92.8	2.56
